# Hydroxyapatite Microspheres as an Additive to Enhance Radiopacity, Biocompatibility, and Osteoconductivity of Poly(methyl methacrylate) Bone Cement

**DOI:** 10.3390/ma11020258

**Published:** 2018-02-07

**Authors:** In-Gu Kang, Cheon-Il Park, Hyun Lee, Hyoun-Ee Kim, Sung-Mi Lee

**Affiliations:** 1 Department of Materials Science and Engineering, Seoul National University, Seoul 08826, Korea; haeronggu@snu.ac.kr (I.-G.K.); iuy64@snu.ac.kr (C.-I.P.); akusaya@snu.ac.kr (H.L.); kimhe@snu.ac.kr (H.-E.K.); 2 Biomedical Implant Convergence Research Center, Advanced Institutes of Convergence Technology, Suwon-si 16229, Korea

**Keywords:** bone cements, poly(methyl methacrylate), hydroxyapatite, radiopaque properties, osteoconductivity

## Abstract

This study demonstrates the utility of hydroxyapatite (HA) microspheres as an additive to enhance the radiopaque properties, biocompatibility, and osteoconductivity of poly(methyl methacrylate) (PMMA)-based bone cements. HA microspheres were synthesized using spray drying. They had well-defined spherical shapes, thus allowing for the production of PMMA/HA composites with a very high HA content (20 vol % and 40 vol %). The uniform distribution of these HA microspheres in the PMMA matrix resulted in a remarkable increase in compressive modulus (*p* < 0.05), while preserving a reasonably high compressive strength. The PMMA/HA bone cements showed much higher radiopacity than PMMA containing BaSO_4_ as the additive. This was attributed to the high HA content up to 40 vol %. In addition, the biocompatibility and osteoconductivity of PMMA/HA bone cements were significantly enhanced compared to those of PMMA bone cements containing BaSO_4_, which were assessed using in vitro tests and in vivo animal experiments.

## 1. Introduction

Poly(methyl methacrylate) (PMMA) bone cement has been widely used as an adhesive material in orthopedics, which can make a bond between implants and bone, or a filler in bone defects caused by osteoporosis or cancer [1,2,3,4,5,6]. In particular, due to its excellent mechanical properties, PMMA bone cement plays a crucial role in load-bearing applications, such as hip and knee replacement implants [7,8]. However, its various strong points in biomaterial applications are limited by several complications. For example, PMMA is a radiolucent polymer which is hard to recognize in X-ray images [9]. For this reason, the interface between the surrounding bone and the implant is imperceptible. In addition, PMMA bone cement is bioinert, which can cause fibrosis (i.e., fibrous encapsulation) around the biomaterial or implant [10], thus leading to poor bone adhesion to the material surface and aseptic loosening of the implant [11,12,13].

Thus, an additive with high radiopacity, such as barium sulfate or zirconium dioxide, is commonly incorporated in bone cements to enhance the radiopaque properties of PMMA bone cements [14]. However, these additives have too low of an osteoconductivity to be used in orthopaedic applications [9,15]. On the other hand, in recent years, hydroxyapatite (HA) has been examined as an additive, particularly due to its excellent biocompatibility and osteoconductivity [16,17,18,19,20]. Basically, it is well known that HA, the major inorganic component of human bone, can direct physicochemical bonding with surrounding bone due to its favorable response to bone when used as a bone substitute for dental and orthopedic applications [21,22]. However, when HA particles are used as an additive in PMMA bone cement, there is an upper limit for HA content (e.g., ~15 wt %), since particles with a large surface area are likely to agglomerate, thus resulting in poor mechanical strength [23]. Thus, it is still a challenge to find new ways of enhancing the HA content in PMMA/HA composite bone cements, in order to induce a more stable attachment and stronger bonding with surrounding tissue, as well as reasonably high mechanical properties for load-bearing applications.

In this study, we examined the potential of HA microspheres as a new type of additive, in order to enhance the biological functions (e.g., biocompatibility and osteoconductivity) of PMMA/HA composite bone cements without sacrificing their high mechanical properties. HA microspheres with well-defined spherical shapes were synthesized using spray drying and then mixed with PMMA/methyl methacrylate (MMA) mixtures. The microstructure, crystalline phases, mechanical properties, and radiopaque properties of the PMMA/HA composites were examined. In addition, their in vitro biocompatibility and in vivo osteoconductivity were evaluated.

## 2. Materials and Methods

### 2.1. HA Microspheres Synthesis

HA microspheres were fabricated by a spray-drying method as described previously [24]. Briefly, 4 g of HA powder (Alfa Aesar Co., Ward Hill, MA, USA) was mixed with 10 mL of ethanol. Then, 1.5 wt % of polyvinyl butyral (PVB; Sigma Aldrich, St. Louis, MO, USA) and 0.1 wt % of oligomer polyester (Hypermer KD-6; UniQema, Everburg, Belgium) were added as a binder and a dispersing agent, respectively. The solution was sprayed out through a nozzle with a diameter of 1 mm under a pressure of 0.15 MPa at 30 °C. The as-formed HA/PVB microspheres were then heat-treated at 500 °C for 10 h to eliminate the organic phases (i.e., binder and dispersing agent), followed by sintering to densify HA at 1350 °C for 2 h. After the process, microspheres were sieved down to 100–500 µm.

### 2.2. Morphology and Microstructure Characterization

The morphology and microstructure of synthesized HA microspheres were evaluated by scanning electron microscopy (SEM; JSM-6360; JEOL Techniques, Tokyo, Japan). For these observations, the surfaces of the HA microspheres were coated with a thin layer of Pt using a sputter coater (MSC-101; JEOL Techniques, Tokyo, Japan).

### 2.3. Crystalline Phases Analysis

The crystalline phases of the HA microspheres were characterized by X-ray diffractometer (XRD; D8 Advance, Bruker Miller Co., Karlsruhe, Germany). They were ground to particulate form for XRD analysis. For comparison purposes, as-received HA powders were also examined.

### 2.4. PMMA/HA Microsphere Composites Preparation

PMMA/HA microsphere composites with various HA contents (5 vol %, 20 vol %, and 40 vol %) were prepared by mixing HA microspheres with PMMA/MMA mixtures. Pre-polymerized PMMA beads (Sunjin Chemical, Ansan, Korea) with a size range of 10–20 µm in diameter were used. An MMA (Yakuri Pure Chemicals Co., Kyoto, Japan) monomer with a purity of 95% was used to complete the polymerization. Benzoyl peroxide (BPO; Sigma Aldrich, St. Louis, MO, USA) and *N*,*N*-dimethyl-*p*-toluidine (DMPT, Sigma Aldrich, St. Louis, MO, USA) were used as initiator and activator of free radical polymerization, respectively. PMMA prepolymer beads and a liquid MMA monomer containing 1 wt % of BPO powder and liquid DMPT activator were hand-mixed at a 2:1 weight ratio. The mixtures were placed into a cylindrical polyester mold with a diameter of 6 mm. The PMMA/HA composites were then cured in a 70% humidified incubator at 37 °C for 24 h to evaporate the residual MMA monomer. For comparison purposes, PMMA bone cements with HA powders or barium sulfate powders (BaSO_4_, Sigma Aldrich, St. Louis, MO, USA) as an additive were also produced in a similar way.

### 2.5. Mechanical Properties Measurement

The mechanical properties of PMMA/HA composites were measured using a universal testing instrument (Instron 5582, Instron Co., Canton, MA, USA). The specimens, 6 mm in diameter and 12 mm in height, were uniaxially compressed at a constant cross-head speed of 1 mm/min and their stress versus strain responses were recorded during the tests [25]. The compressive strength and modulus were calculated from the stress versus strain curve of the specimens. For comparison purposes, PMMA bone cements with barium sulfate as an additive were also tested. Five samples were tested to obtain mean and standard deviation.

### 2.6. In Vitro Biocompatibility Evaluation

The in vitro biocompatibility of PMMA/HA composites was evaluated in terms of the attachment, proliferation, and differentiation of pre-osteoblast cells (MC3T3-E1). For comparison purposes, PMMA bone cements with barium sulfate as an additive were also tested. Specimens with a diameter of 12 mm and a height of 2 mm were sterilized by 70% ethanol for 1 h, autoclaved, and UV-treated for 24 h. MC3T3-E1 cells (ATCC, CRL-2593; Rockville, MD, USA) were seeded onto each sample at a cell density of 3 × 10^4^ cells/mL. They were then cultured in a Minimum Essential Medium (α-MEM, Welgene Co., Ltd., Gyeongsan, Korea) supplemented with 5% fetal bovine serum (FBS, Life Technologies, Inc., Waltham, MA, USA) and 1% antibiotics (100 U/mL penicillin and 100 μg/mL streptomycin, GIBCO, Grand Island, NY, USA) in a humidified incubator at 37 °C with 5% CO_2_. Cellular morphology was observed by SEM after 6 h of incubation. For SEM observation, cells were fixed with 2.5% glutaraldehyde for 10 min and dehydrated in graded ethanol (75%, 95% and 100%). Sequentially, they were immersed in hexamethyldisilazane for 10 min and air dried. The proliferation rate of the cells was examined by methoxyphenyl tetrazolium salt (MTS) assay (CellTiter 96 Aqueous One Solution, Promega, Madison, WI, USA) after 3 days and 5 days of culturing. The amount of formazan proportionate to the number of living cells was determined based on light absorbance value at a wavelength of 490 nm on a micro-reader (Model 550; Biorad, Hercules, CA, USA). The cell differentiation level was determined using an alkaline phosphatase (ALP) activity test. To detect the differentiation of osteoblastic cells, 10 mM β-glycerophosphate (β-GP) and 50 μg/mL ascorbic acid (AA) were included with the culture medium. After 10 days of culturing, the produced amount of p-nitrophenol (pNP) was colorimetrically measured based on light absorbance at a wavelength of 405 nm on a micro-reader. With ALP present in the medium, p-nitrophenyl phosphate (pNPP) is converted to pNP. Therefore, the rate of pNP produced was proportional to the ALP activity.

### 2.7. In Vitro Radiopaque Properties Evaluation

The radiopaque properties of the PMMA/HA composites were examined by micro-CT (Skyscan 1173 X-ray μ-tomography System; Skyscan, Kontich, Belgium) in vitro and compared to those of the PMMA bone cements containing barium sulfate as an additive.

### 2.8. In Vivo Animal Tests

PMMA/HA composite with an HA content of 40 vol % was chosen as the implanting material for the in vivo animal tests. For comparison purposes, PMMA bone cements containing barium sulfate as an additive were also tested. The in vivo animal tests were performed according to the protocol approved by the Institutional Animal Care and Use Committee of Genoss (no. 1703-05). A total of three New Zealand white male rabbits (12 weeks old, average weight of 3 kg) were used for these tests. These animals were weighed at the beginning and the end of the experiment. All rabbits had a normal diet. They were cared for under the same conditions. Specimens used for the in vivo tests were prepared using powder and liquid components separately. They were mixed right before implantation. Powder components were sterilized by γ-ray irradiation while liquid components were purified by filtering.

**Figure 1:**
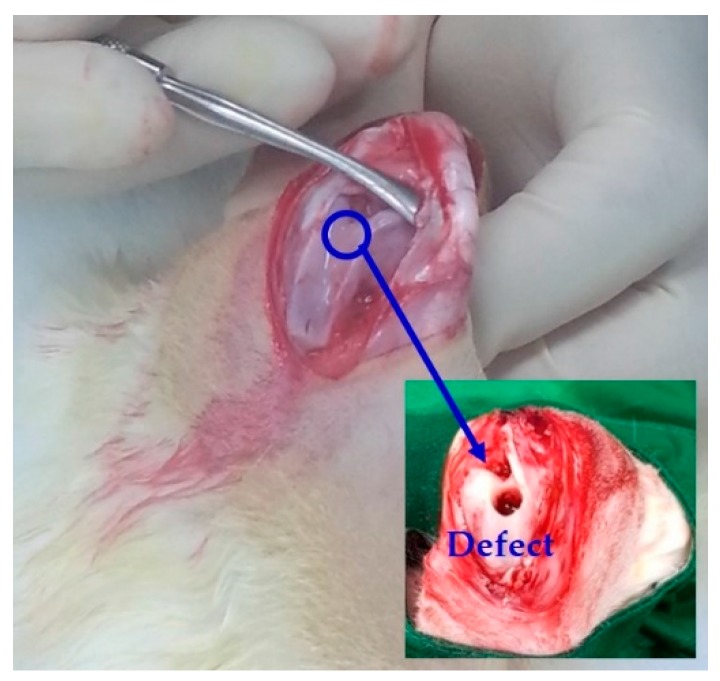
Optical image of the defect created in the femoral condyle of the rabbit.

The PMMA/HA composite and PMMA bone cements were implanted into defects created in the femoral condyles of the rabbits. Surgical sites were shaved and the skin was treated with a surgical prep solution containing 10% povidone-iodine (Betadine, Mundipharma Korea, Seoul, Korea). The surgery was performed under conditions of general anesthesia with 0.1 cc of 2% Xylazine HCl (Rompun, Bayer Korea, Seoul, Korea), 0.2 cc of Tiletamine HCl (Zoletil, Virbac lab, Carros, France), and Lidocaine (Yuhan Corporation, Seoul, Korea). An incision of approximately 4 cm was made at each site of implant placement. A trephine bur with a diameter of 6 mm was used to make defects on each side of the femoral condyle (Figure 1). PMMA/HA composite paste was injected into the femoral defect on one side and PMMA paste containing barium sulfate was injected into the femoral defect on the other side. A total of three rabbits were used for osteoconductivity analyses. Wounds were sutured using Surgisorb (Samyang Ltd., Seoul, Korea).

Immediately after the surgery, the radiological property was monitored by a portable X-ray device. Gentamicin (0.1 mg/kg) was immediately injected after the operation and every 24 h for three days. Animals were monitored daily for any adverse reaction. At 4 and 8 weeks following the implantation, animals were sacrificed by asphyxiation with carbon dioxide. Rabbit femora were harvested. The tissue from the implant site was collected and fixed in 10% neutral buffered formalin.

### 2.9. In Vivo Radiopaque Properties Evaluation

The radiopaque properties of the PMMA/HA composite and PMMA containing barium sulfate were evaluated by a portable X-ray device (iRay D4; Dexcowin, Los Angeles, CA, USA) in vivo after implantation.

### 2.10. In Vivo Osteoconductivity Evaluation

For histological analysis, the extracted tissues from the PMMA/HA composite and PMMA containing barium sulfate were embedded in resin (Technovit 7200 VLC, Kulzer, Hanau, Germany). Thin sections of blocks at a thickness of 40 µm were prepared using an Exakt cutting and grinding system (Exakt cutting and grinding system, Exakt, Oklahoma City, OK, USA). They were then stained with Goldner’s trichrome. Optical microscopic images of samples were prepared using Axioskop microscopy (Olympus BX51, Olympus, Tokyo, Japan). In the optical images, the mature bone matrix was stained green [21]. The volumes of bone tissues newly formed around and within the PMMA and PMMA/HA composite were calculated on the basis of micro-CT analyses.

### 2.11. Statistical Analysis

Data are shown as mean and standard error deviation (SED). Statistical analysis was carried out using one-way analysis of variance (ANOVA). *P* values of less than 0.05 were considered statistically significant.

## 3. Results and Discussion

### 3.1. Morphology and Microstructure of HA Microspheres

**Figure 2:**
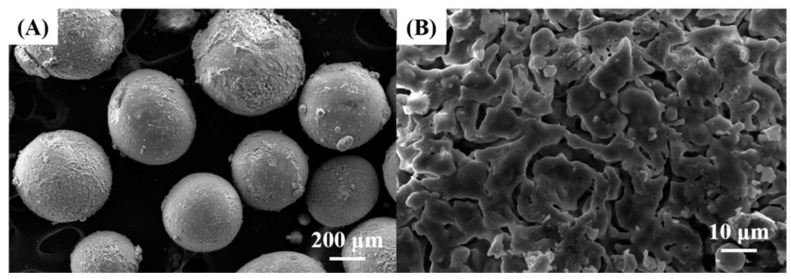
Representative SEM images of synthesized hydroxyapatite (HA) microspheres displaying (**A**) well-defined spherical shape and (**B**) relatively rough surface.

HA microspheres were successfully produced using the spray-drying method, as shown in Figure 2A,B. A well-defined spherical shape without noticeable defects, such as collapse in shape or cracking within the microsphere, was obtained after sintering at 1350 °C for 2 h. This finding suggests that PVB polymer used as the binder could effectively hold HA particles after spraying at 30 °C, thus allowing for the formation of spherical microspheres with a reasonably high green strength. In addition, the PVB binder could be completely removed via heat treatment at 500 °C for 10 h prior to sintering without destroying the initial spherical shape of the microsphere. HA microspheres with controlled sizes in the range of 100–500 μm were obtained using a conventional sieving process (Figure 2A), in order to provide good mixing with PMMA bone cements. However, these HA microspheres had a rough and porous microstructure (Figure 2B) due to the use of a relatively low HA content in HA suspension (i.e., 30 vol %).

### 3.2. Crystalline Phases of HA Microspheres

**Figure 3:**
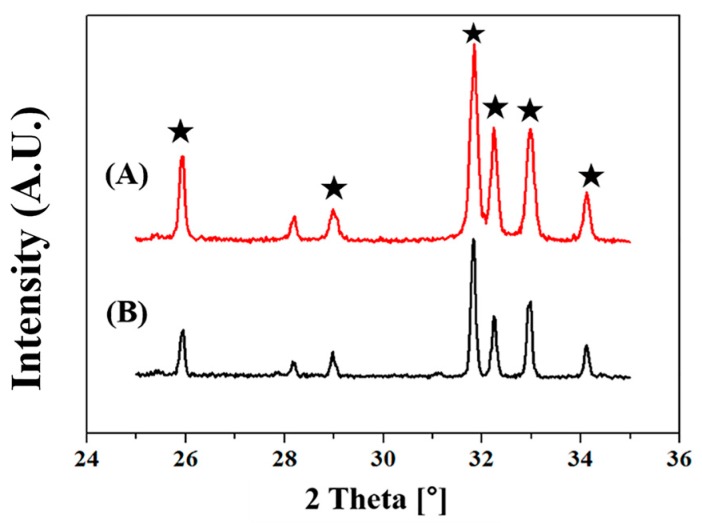
Representative XRD patterns of (**A**) as-received HA powders and (**B**) HA microspheres synthesized using spray drying (★: HA phase).

The crystalline phases of the HA microspheres were evaluated by XRD, since they would strongly affect biological functions in vivo (e.g., biocompatibility, bioactivity, and osteoconductivity) when used as an additive for PMMA bone cements [26,27,28,29]. For comparison purposes, as-received HA powders were also examined. Figure 3A,B display representative XRD patterns of the HA powders and HA microspheres, respectively. Basically, the crystalline phases of the HA microspheres did not change even after sintering at 1300 °C. That is, only peaks corresponding to the crystalline HA phase were observed without any secondary peaks (Figure 3B). This finding suggests that the spray-drying method used in this study can synthesize HA microspheres without altering their crystalline phases, and thus excellent osteoconductivity can be preserved [23,30,31].

### 3.3. Mixing Behavior of HA Microspheres with PMMA Bone Cements

**Figure 4:**
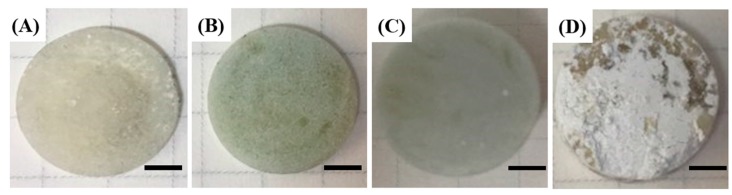
Representative optical images of poly(methyl methacrylate) (PMMA)/HA composites produced using the HA microspheres with various HA contents of (**A**) 5 vol %; (**B**) 20 vol %; (**C**) 40 vol %; and (**D**) composite produced using the HA powders with an HA content of 20 vol % (Scale bar = 3 mm).

The mixing behavior of HA microspheres with PMMA bone cements was evaluated, since it would strongly affect the mechanical properties, radiopaque properties, and osteoconductivity of PMMA/HA composites [32]. For comparison purposes, HA powders as an additive were also tested. Figure 4A–D show representative optical images of PMMA/HA composites produced using HA microspheres with various HA contents (5 vol %, 20 vol %, and 40 vol %) and PMMA/HA composite produced using HA powders (20 vol %). When HA powders were used as an additive, severe agglomeration of HA particles with a rough surface was observed when HA content was at 20 vol % (Figure 4D). This limited mixing behavior is due to the relatively high surface area of HA powders, as is often the case with inorganic powder-added PMMA bone cements [32]. On the other hand, when HA microspheres were used as an additive, a smooth surface morphology without any signs of agglomeration of HA microspheres was observed for all PMMA/HA composites. It is worth noting that a very high HA content of 40 vol % (80 wt %) could be uniformly mixed with PMMA bone cement (Figure 4C). This is one of the most striking advantages of HA microspheres with a well-defined spherical shape as an additive. For example, high HA contents would significantly enhance the osteoconductivity of PMMA/HA bone cements, thus providing strong attachment of implants to surrounding bones. 

### 3.4. Microstructure of PMMA/HA Composites

**Figure 5:**
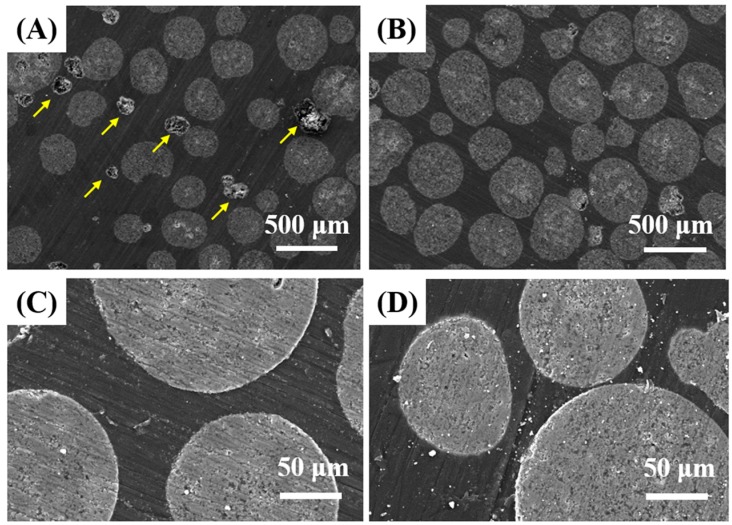
Representative SEM images of PMMA/HA composites produced using the HA microspheres with various HA contents of 20 vol % (**A**,**C**) and 40 vol % (**B**,**D**), showing distribution of the HA microspheres in the PMMA matrix (**A**,**B**) and interfacial bonding between the HA microspheres and PMMA (**C**,**D**). Yellow arrows indicate voids formed during the grinding process for SEM observations.

The microstructure of PMMA/HA composites, including the dispersion of HA microspheres in the PMMA matrix and interfacial bonding between HA microspheres and PMMA, was evaluated by SEM. Figure 5A–D show representative SEM images of PMMA/HA composites with various HA contents (20 vol % and 40 vol %). For both composites, HA microspheres were uniformly distributed in the PMMA matrix, while preserving their original spherical shapes (Figure 5A,B). Some voids, indicated by yellow arrows, were formed due to the pull-out of HA microspheres during the grinding process for SEM observations (Figure 5A). Thus, HA debris detached from the HA microspheres appear bright white. However, no noticeable interfacial delamination between HA particles and PMMA was observed for these composites (Figure 5C,D). This finding suggests that the use of HA microspheres as an additive for PMMA bone cements can enable the achievement of very high HA contents (up to 40 vol %) that would be unobtainable using HA powders, thus providing significantly enhanced mechanical, biological, and radio-opacifying functions.

### 3.5. Mechanical Properties of PMMA/HA Composites

**Figure 6:**
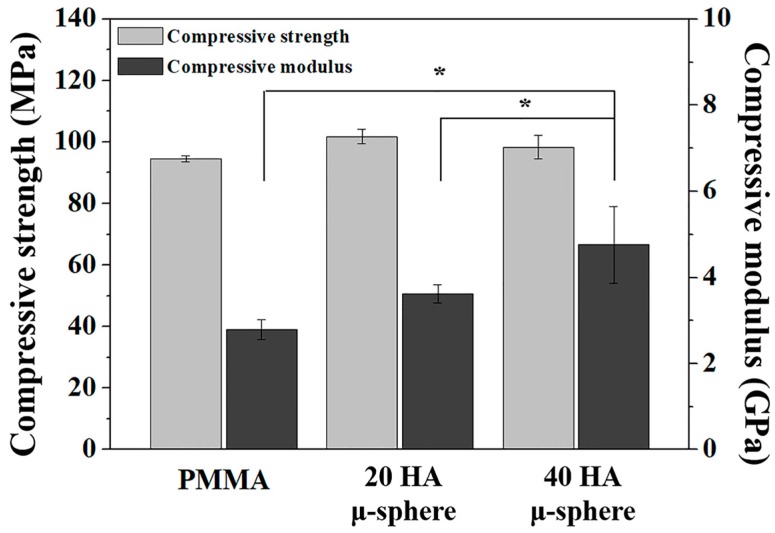
Compressive strengths and compressive modulus of PMMA/HA composites with various HA contents of 20 vol % (20 HA μ-sphere) and 40 vol % (40 HA μ-sphere) and PMMA bone cement containing barium sulfate (PMMA) (* *p* < 0.05).

The mechanical properties of PMMA/HA composites were evaluated using compressive strength tests and compared to those of pure PMMA bone cement without HA addition. Figure 6 displays compressive strengths and compressive modulus of PMMA/HA composites with various HA contents (20 vol % and 40 vol %) and PMMA bone cement. Interestingly, the PMMA/HA composites showed similar compressive strengths in the range of 90–100 MPa, comparable to that of the PMMA bone cement. No statistically significant differences between these groups were observed. These high compressive strengths could be achieved through the uniform distribution of HA microspheres in the PMMA matrix (c.f. Figure 4B,C). It should be noted that when HA particles are used as an additive, they are likely to agglomerate (c.f. Figure 4D), which would act as rack initiation sites, thus causing severe reduction in mechanical strength [32,33]. On the other hand, the compressive modulus was increased from 3.62 ± 0.20 GPa to 4.76 ± 0.89 GPa with an increase in HA content from 20 to 40 vol %. Interestingly, the compressive modulus of 40 vol % HA was significantly higher than that of the PMMA bone cement (2.79 ± 0.22 GPa, *p* < 0.05). Such enhancement is mainly attributed to the presence of HA phase that has much higher stiffness than PMMA [7]. However, it should be noted that the compressive moduli (~3.62–4.76 GPa) of the PMMA/HA composites are still comparable to those (~3.5–8.6 GPa) of natural bones. This would mitigate the stress-shielding effect, thus providing long-term stability in vivo [34,35,36,37,38].

### 3.6. In Vitro Biocompatibility of PMMA/HA Composites

**Figure 7:**
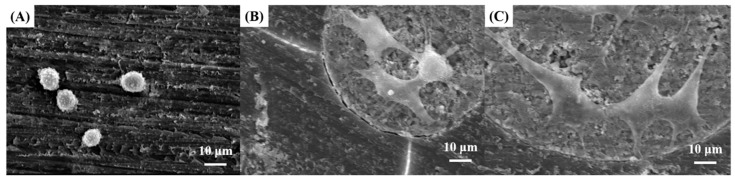
Representative SEM images of the MC3T3-E1 on (**A**) PMMA with barium sulfate as an additive; PMMA/HA composites with various HA contents of (**B**) 20 vol % and (**C**) 40 vol % after 6 h of culturing.

The effect of the addition of HA microspheres on the in vitro biocompatibility of PMMA/HA composites was evaluated in terms of the attachment, proliferation, and differentiation of pre-osteoblasts (MC3T3-E1). For comparison purposes, PMMA bone cement with barium sulfate as an additive was also tested. Figure 7A–C show representative SEM images of MC3T3-E1 on the PMMA and PMMA/HA composites with various HA contents (20 vol % and 40 vol %) after 6 h of culturing. The PMMA revealed cells with a round shape, suggesting limited biocompatibility (Figure 7A). On the other hand, the PMMA/HA composites showed that cells adhered to and actively spread, particularly on the surface of HA microspheres (Figure 7B,C). In addition, cells showed filopodia-like extension and branching owing to the excellent biocompatibility of the HA microspheres [21,22,39]. However, it should be noted that the PMMA/HA composite with a higher HA content of 40 vol % can provide a larger surface area for cell attachment.

**Figure 8:**
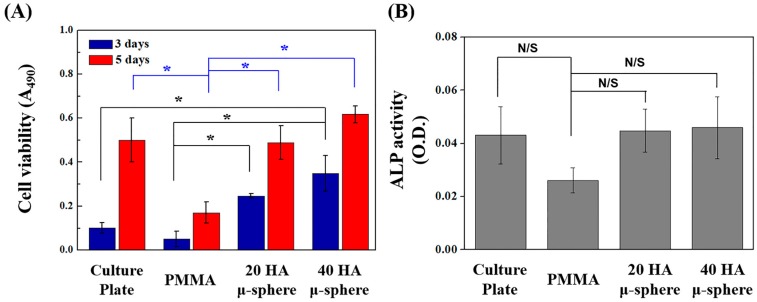
Representative (**A**) levels of cell viability and (**B**) alkaline phosphatase (ALP) activity on tissue culture plate (TCP), PMMA bone cement containing barium sulfate (PMMA), and PMMA/HA composites with various HA contents of 20 vol % (20 HA μ-sphere) and 40 vol % (40 HA μ-sphere) (* *p* < 0.05, N/S: not statistically significant).

The cell proliferation and differentiation of the MC3T3-E1 cells were evaluated by the MTS and ALP measurements, respectively. For comparison purposes, tissue culture plate (TCP) as a control was also tested. Figure 8A,B show levels of cell viability and ALP activity, respectively, observed for the PMMA containing barium sulfate, PMMA/HA composites with various HA contents (20 vol % and 40 vol %), and TCP. After 3 days of cell culturing, the PMMA/HA composites with various HA contents (20 vol % and 40 vol %) showed significantly higher cell viability (*p* < 0.05) than PMMA and even tissue culture plate used as the control (Figure 8A). However, all samples showed that cell viability was increased remarkably with an increase in culturing period, indicating that they have good biocompatibility in vitro. After 5 days of cell culturing, the PMMA/HA composite with the highest HA content of 40 vol % showed a significantly higher cell viability (*p* < 0.05) than the PMMA/HA composite with an HA content of 20 vol %. This is attributed to the fact that a higher fraction of HA microspheres can be exposed to the surface that can facilitate cell adhesion and proliferation. Similarly, the PMMA/HA composites showed higher levels of ALP activity after 7 days of cell culturing (Figure 8B). However, no statistically significant difference between the PMMA/HA composites with various HA contents (20 vol % and 40 vol %) was observed. These findings suggest that the in vitro biocompatibility of PMMA bone cements can be significantly enhanced by using bioactive HA microspheres as an additive.

### 3.7. Radio-Opacifying Properties of PMMA/HA Composites

**Figure 9:**
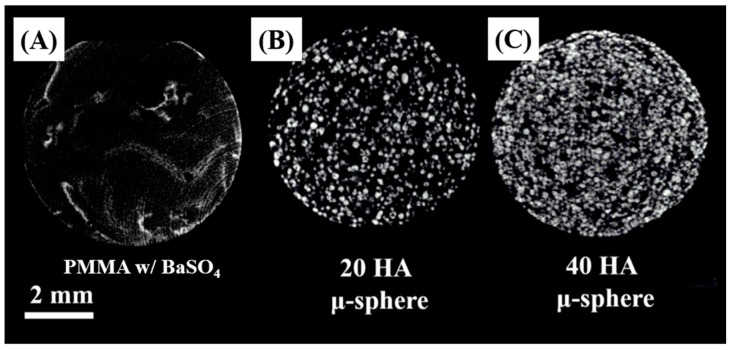
Representative μ-CT images captured from (**A**) PMMA containing barium sulfate; PMMA/HA composites with various HA contents of (**B**) 20 vol % and (**C**) 40 vol %.

For clinical applications, bone cements should have reasonably high radiopacity, so that the bone healing process can be monitored in situ using medical imaging techniques. To clarify this issue, the radiopaque properties of the PMMA/HA composites were examined using both micro-CT in vitro and X-ray in vivo. As reference, a PMMA bone cement containing BaSO_4_ particles (2.5 vol %) as a radio-opacifying additive with compositions similar to those of commercial PMMA bone cements was also tested. Figure 9A–C show representative μ-CT images captured from the PMMA and PMMA/HA composites with various HA contents (20 vol % and 40 vol %). The PMMA displayed a relatively low intensity of signals due to a small amount of BaSO_4_ particles (Figure 9A). On the other hand, the PMMA/HA composites could be clearly detected using micro-CT—bright contrast areas that appeared from the HA microspheres with high radiopacity (Figure 9B,C). In addition, the intensity of signals was remarkably increased with an increase in HA content. It should be noted that such high radiopacity can be achieved through the incorporation of a large amount of HA microspheres into PMMA/HA composites. It would be unobtainable when HA powders are used as an additive.

**Figure 10:**
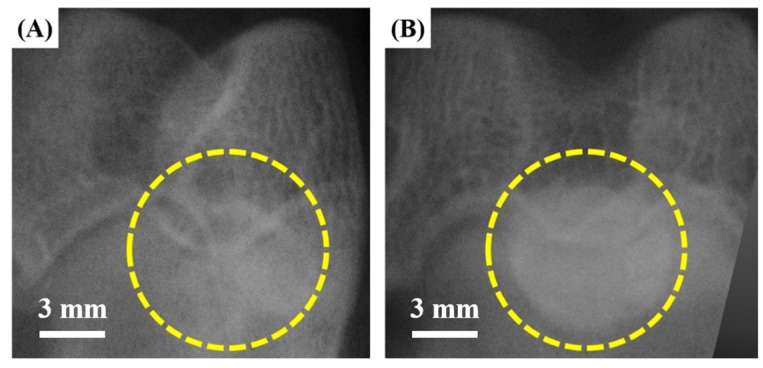
Representative X-ray images captured from (**A**) PMMA containing barium sulfate and (**B**) PMMA/HA composite with an HA content of 40 vol %, immediately after implantation into defects created in the femoral condyles of rabbits. Dashed lines indicate the bone cements in the defects.

Figure 10A,B show representative X-ray images captured from the PMMA containing barium sulfate and PMMA/HA composite with an HA content of 40 vol %, respectively, immediately after implantation into defects created in the femoral condyles of rabbits. Dashed lines indicate the bone cements in the defects. The PMMA bone cement could be detected in vivo due to the presence of radiopaque BaSO_4_ particles; however, the intensity of signals was relatively low (Figure 10A). On the other hand, the PMMA/HA composite displayed a much stronger intensity of signals (Figure 10B), owing to the uniform distribution of radiopaque HA microspheres. Thus, it is reasonable to suppose that the high radiopaque property of the PMMA/HA composite would allow for more accurate monitoring of the bone healing process in vivo.

### 3.8. Osteoconductive Properties of PMMA/HA Composites

**Figure 11:**
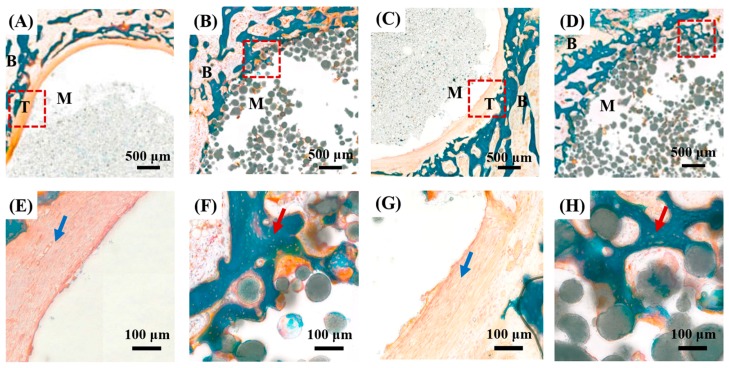
Representative histological cross-sectional images of PMMA containing barium sulfate (**A**,**E**,**C**,**G**) and PMMA/HA composite with an HA content of 40 vol % (**B**,**F**,**D**,**H**) after 4 weeks (**A**,**E**,**B**,**F**) and 8 weeks (**C**,**G**,**D**,**H**) of implantation (B: bone, T: soft tissue, M: material). Blue and red arrows indicate soft tissue and new bone, respectively.

The osteoconductivity of the PMMA/HA composite, which is one of the most critical requirements to securely fix implants and surrounding bones, was evaluated using in vivo animal experiments. PMMA bone cement containing BaSO_4_ particles was used as reference. Figure 11A–H display representative histological cross-sectional images of the PMMA containing barium sulfate and PMMA/HA composite after 4 and 8 weeks of implantation. For the PMMA, soft tissues were formed at the interfaces between the bone cement and surrounding bones (Figure 11A,E). In addition, no structural, direct bonding between the PMMA and surrounding bones was observed even after 8 weeks of implantation (Figure 11C,G). This finding indicates that PMMA bone cements containing BaSO_4_ particles as an additive lack osteoconductive capability, as is often the case with conventional PMMA bone cements [11,40]. On the other hand, for the PMMA/HA composite, new bone tissues were vigorously created at the interfaces (Figure 11B,F), suggesting its outstanding osteoconductivity [11,31]. Interestingly, new bone tissues could penetrate into the PMMA/HA composite bone cement after 8 weeks of implantation, marked by red arrows (Figure 11D,H). This finding suggests that the incorporation of biocompatible, bioactive, and osteoconductive HA microspheres can significantly enhance the biological functions of PMMA-based composite bone cements.

**Figure 12:**
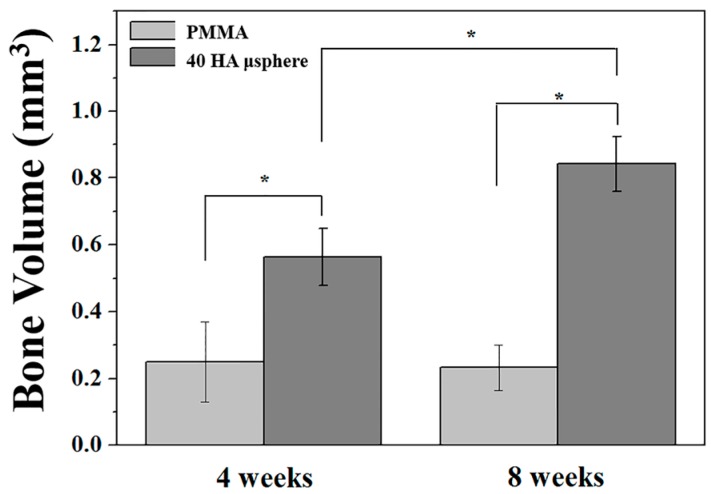
Volumes of bone tissues newly formed around and within PMMA and PMMA/HA composite with an HA content of 40 vol % after 4 and 8 weeks of implantation (* *p* < 0.05).

The volumes of bone tissues newly formed around and within the PMMA and PMMA/HA composite were calculated on the basis of micro-CT analyses, as shown in Figure 12. As expected, the PMMA/HA composite showed much higher bone volume than the PMMA (*p* < 0.05). In addition, the bone volume increased remarkably (*p* < 0.05) with an increase in implantation period for the PMMA/HA composite. These findings suggest that the use of HA microspheres as an additive can significantly enhance the osteoconductivity and bone regeneration ability of PMMA bone cements, thus providing excellent mechanical functions and stability when used as bone cements.

## 4. Conclusions

PMMA/HA composite bone cements with very high HA contents (20 vol % and 40 vol %) were successfully produced using HA microspheres as an additive. Owing to their spherical shapes and controlled sizes (100–500 μm), HA microspheres could be uniformly dispersed in the PMMA matrix with strong interfacial bonding. This allowed the PMMA/HA composites to have high mechanical properties comparable to those of pure PMMA—compressive strength of ~98 MPa and compressive modulus of ~4.76 GPa for the composite with an HA content of 40 vol %. In addition, the PMMA/HA composites showed much higher radiopacity than conventional PMMA bone cements owing to a high content of radiopaque HA phase, thus allowing for more accurate monitoring of the bone healing process in vivo. The use of HA phase as an additive provided significantly enhanced in vitro biocompatibility and in vivo osteoconductivity. These findings suggest that HA microspheres can be effectively used as an additive to enhance the radiopaque properties, biocompatibility, and osteoconductivity of poly(methyl methacrylate) (PMMA)-based bone cements, as well as high mechanical properties for orthopedic applications.
